# Asthenic Amaurosis Cured by the Use of Convex Spectacles

**Published:** 1843-06

**Authors:** 


*Asthenic Amaurosis cured by the use of convex Spectacles.*—Taking a hint from certain quack vendors of spectacles, Dr. Cunier, of Brussels, has made trial of the effects of convex glasses, in what he terms *cases of simple anæsthesia of the retina*, and with a considerable share of success. The influence of the light, directed upon the retina by such glasses, appears beneficially to excite the sunken sensibility of the optic nerve.

A lady, whose case he relates, could not for eight years without difficulty distinguish with her left eye the large characters forming the title of a newspaper; could not tell the hour on the clock, unless her eye was within two inches of the hands, nor distinguish the feature nor the figure of a person at the distance of two feet. If the right eye was covered, the left pupil became dilated, and remained so.

Glasses, whether concave or convex, ought always to be distinguished by their focal lengths, and not by numbers. As Dr. Cunier employs the latter mode, we are at a loss to know the precise power of No. 3, with which the patient could read a large type with her left eye, but probably it was a convex lens of three inches focus. After exercising the eye with this lens for some minutes, vision became confused and the head painful, so that the patient was obliged to desist. Next day she could with No. 3½, and on the third day with No. 4. The duration of this kind of exercise was gradually lengthened, and glasses of longer and longer focus employed. By the tenth day, the patient read the hour on the clock at the distance of 22 inches, and recognised individuals at double that distance. By the seventeenth day of the treatment, No. 24 was employed, and the patient could read small type. After two months use of No. 24, the sight of the left eye was as good as that of the right.

Several other successful cases are related by Dr. Cunier.—*B. and F. Med. Rev.*, from *Annales d'Oculistique*, May, 1842.

